# Primary plasmacytoma of the testicle: a case report

**DOI:** 10.1186/1752-1947-5-494

**Published:** 2011-10-03

**Authors:** Claudia Berrondo, Timothy E Gorman, Ronald L Yap

**Affiliations:** 1 Dartmouth Medical School, 1 Rope Ferry Road, Hanover, NH 03755, USA; 2 Concord Hospital Center for Urologic Care, 246 Pleasant Street, Memorial Building G-2 Concord, NH 03301, USA

## Abstract

**Introduction:**

Extramedullary plasmacytoma is a rare plasma cell neoplasm. Plasmacytomas are most commonly found in the head and neck region, but can occur in many other locations. They rarely occur in the testis, and are commonly associated with concurrent multiple myeloma at the time of diagnosis. Isolated plasmacytoma of the testis is exceedingly rare, with few cases reported in the literature.

**Case presentation:**

A 72-year-old Caucasian man presented with a painless testicular mass treated by orchiectomy. The mass was determined to be plasmacytoma on pathological examination. At the time of diagnosis, our patient did not have multiple myeloma, and is currently undergoing chemotherapy for treatment of his disease.

**Conclusion:**

Isolated plasmacytoma of the testicle is a rare cause of testicular mass, and is seldom reported in the literature. Patients with this disease require careful monitoring because of their high risk of progression to multiple myeloma. The diagnosis of testicular plasmacytoma can be challenging for primary care doctors and urologic specialists. This condition should be in the differential diagnosis in elderly men.

## Introduction

Patients presenting with extramedullary plasmacytoma (EMP) often present with signs and symptoms of diffuse disease. These patients are generally diagnosed with multiple myeloma at the time of presentation. EMP can occur in many different locations in the body. The most common anatomic site for the disease is the head and neck region, particularly of the respiratory or gastrointestinal tracts. Occasionally, these tumors are located in other organ systems including lymph nodes, liver, skin and, very rarely, the testis [1-3]. Cases of isolated testicular plasmacytoma are extraordinarily rare, with few cases reported in the literature to date [4]. These patients have a high rate of progression to disseminated disease, and they require close monitoring after appropriate treatment [1,2,5]. We discuss a case of isolated testicular plasmacytoma in a 72-year-old patient with ensuing progression to multiple myeloma.

## Case presentation

**Figure 1:**
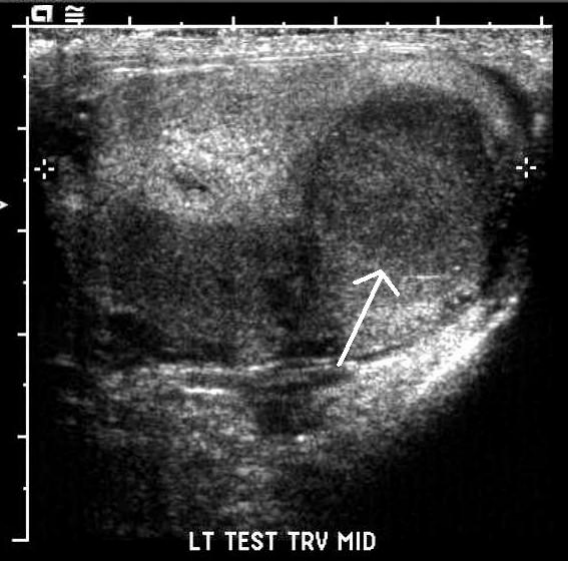
**Ultrasound image of the left testicle**. Arrow annotates abnormal mass lesion within testicular parenchyma.

**Figure 2:**
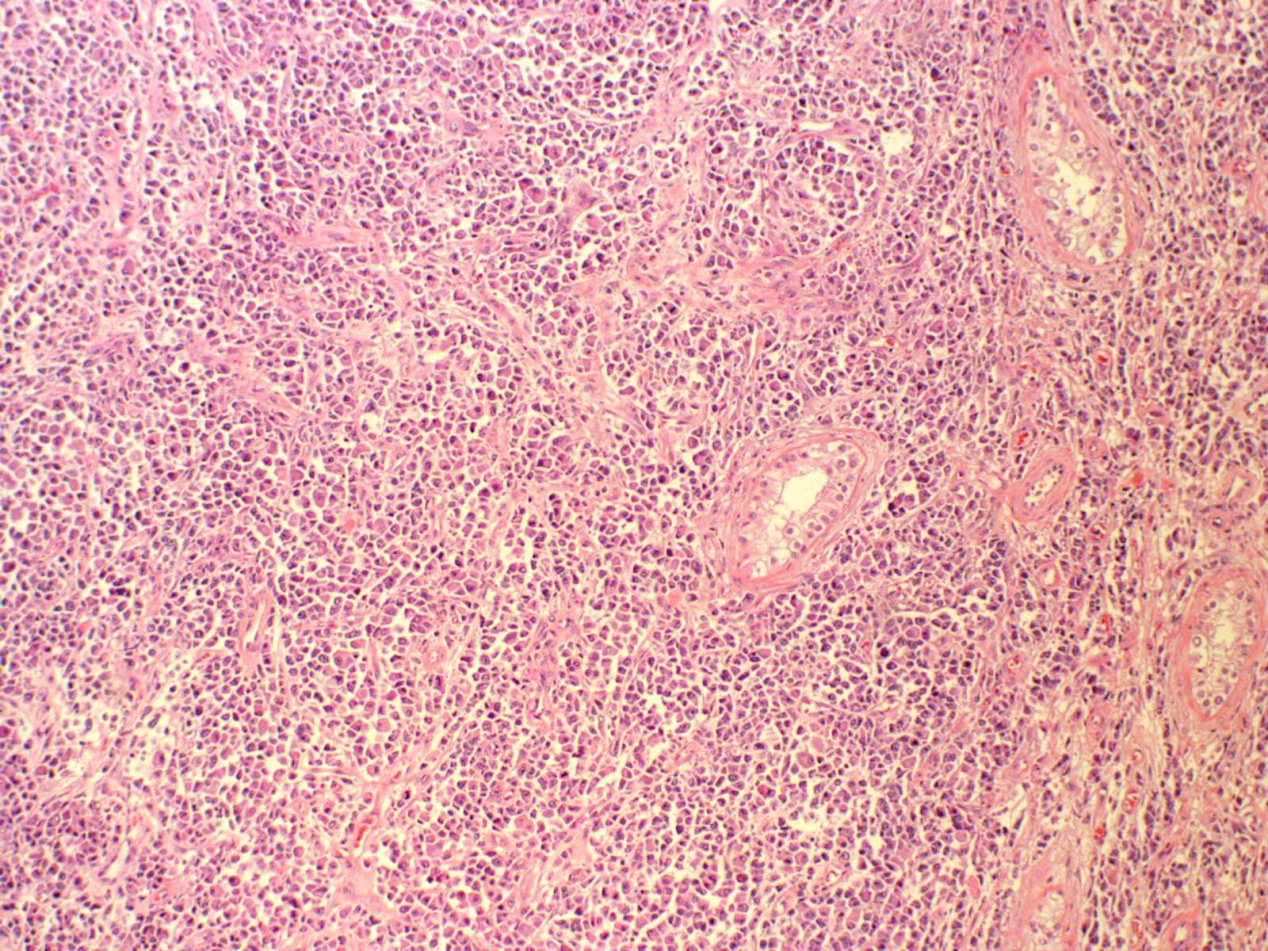
**Hematoxylin and eosin stain of a section of the tumor removed from the left testicle**.

A 72-year-old Caucasian man presented to clinic complaining of a painless left testicular mass. He had no associated bone pain or weight loss. A physical exam revealed a nontender 3 by 5 cm indurated mass in his left testicle. A comprehensive metabolic panel and complete blood count (CBC) revealed a total protein of 8.3, but were otherwise normal. Tumor markers (α-fetoprotein, lactate dehydrogenase, β-human chorionic growth hormone) were negative. On a scrotal ultrasound, the mass appeared multilobar and heterogeneous, thus raising concern for malignancy (Figure 1). Our patient underwent an uncomplicated left inguinal radical orchiectomy. Pathologic evaluation of the testicular mass demonstrated plasmacytoma (Figure 2). Serum protein electrophoresis (SPEP) showed an immunoglobulin A (IgA) level of 2631 mg/dL indicative of monoclonal gammopathy of undetermined significance (MGUS). A skeletal survey was negative for coexisting lesions. A bone marrow biopsy was negative for clonal plasma cells. Our patient continued follow-up with medical oncology and subsequently developed metastatic disease two and a half years later, detected by skeletal survey. He is currently being treated with the chemotherapeutic agent bortezomib with dexamethasone and zoledronic acid.

## Discussion

Plasma cell neoplasms are divided into two different categories: multiple myeloma and solitary plasmacytoma. Solitary plasmacytomas are most commonly found in the bone, however they can also be extramedullar. 90% of all EMPs are found in the head and neck region, particularly the upper respiratory and digestive tracts. Other locations include the gastrointestinal tract, central nervous system, skin and, rarely, the testis. EMPs account for only 3% of plasma cell malignancies. The mean age of diagnosis is 55 to 60 years, with a male to female ratio of two to one [1-3]. The diagnosis of EP requires many diagnostic studies including CBC with differential and smear, complete metabolic panel, SPEP with immunofixation of immunoglobulins, biopsy of the lesion, bone aspiration and biopsy, and metastatic bone survey by positron emission tomography (PET) with computed tomography (CT) or magnetic resonance imaging (MRI). By definition, patients with EMP cannot have symptoms of multiple myeloma including anemia, hypercalcemia, or renal insufficiency. The lesion should have evidence of clonal plasma cells, and the bone marrow biopsy must contain no clonal plasma cells. Some patients may have small amounts of monoclonal protein, usually IgA, in the serum or urine. The marrow of some patients may have up to 10% clonal plasma cells. These patients are considered to have both EMP and MGUS. These patients have higher risk of progressing to multiple myeloma [3,6]. The treatment of these tumors is either radiation therapy or surgical resection. Adjuvant radiation or chemotherapy does not improve the outcome. In patients with incomplete resection, local radiation is the best treatment. Less than 10% of patients develop local recurrence. These patients have high rates of progression to multiple myeloma, up to 15% [7]. The overall 10-year survival for patients with EMP is 70% [3].

Isolated testicular plasmacytoma accounts for only 0.03-0.1% of all testicular tumors [1,7]. The vast majority of patients with testicular plasmacytoma either have disseminated disease at the time of diagnosis, or develop disseminated disease later in life [1,2,5]. This case is therefore unusual due to the primary nature of the plasmacytoma within the testis. The age of diagnosis ranges from 26 to 83 years of age, although the mean age of diagnosis in 55 to 60 years old [4]. The incidence of plasmacytoma also increases with age [8]. Patients commonly present with a firm testicular mass, which may or may not be tender. Patients with disseminated disease may also present with symptoms of multiple myeloma such as back pain. On gross examination, the tumors are soft, fleshy, and white or grey in color [4]. On ultrasound, plasmacytoma of the testicle can be either homogeneous or heterogeneous, and typically hypoechoic. Hyperemia on Doppler imaging has also been observed in these tumors, although hyperemia is also characteristic of many types of testicular tumors [5,9]. On microscopic examination, the tumor appears as sheets of atypical plasma cells with varying degrees of differentiation [5]. Plasmacytomas can be mistaken for other types of tumors, including seminoma, lymphoma and metastatic melanoma [2,4]. In order to make accurate diagnosis, immunologic staining for CD 138, CD 79a and monoclonal antibody VS 38 can be used [4]. Additionally, immunostaining will reveal IgG, IgD or IgA light chains; IgA being the most common [7]. The treatment of choice for testicular plasmacytoma is radical orchiectomy. In addition, these tumors are highly radiosensitive so a combination of surgery and radiation can be implemented. For patients with residual disease after surgery, or recurrent or refractory disease, radiation can be used as well [4]. The overall prognosis for patients with testicular plasmacytoma is poor, with high rates of progression to multiple myeloma. Because of the high rates of progression, these patients require close monitoring and long-term surveillance. There are no established guidelines as to which tests are appropriate for surveying for metastatic disease, or for the frequency or duration of surveillance. A common approach includes a combination of periodic history and physical exam, laboratory tests (urine and serum protein electrophoresis with immunofixation, CBC, serum creatinine, serum calcium) and imaging such as PET with or without CT or MRI at lengthening intervals.

## Conclusion

EMP is a rare form of plasma cell neoplasm. This tumor can present in many locations in the body, the testicle being one of the rarer sites. When plasmacytomas occur in the testis, the diagnosis can be difficult. Plasmacytomas often resemble other more common causes of testicular mass, and require multiple diagnostic tests for accurate diagnosis. Plasmacytomas often present concurrently with multiple myeloma, but can present as an isolated tumor. Patients with isolated plasmacytoma have high rates of progression to multiple myeloma later in life. For this reason, it is important to accurately diagnose plasmacytoma and survey these patients appropriately for progression to disseminated disease. Plasmacytoma of the testicle is exceedingly rare, but an important disease to consider in patients presenting with testicular mass, particularly an elderly patient.

## Consent

Written informed consent was obtained from the patient for publication of this case report and any accompanying images. A copy of the written consent is available for review by the Editor-in-Chief of this journal.

## Competing interests

The authors declare that they have no competing interests.

## Authors' contributions

CB wrote and edited the manuscript. TG created pathologic images, read and approved the manuscript. RY provided patient care, designed the study and wrote and edited the manuscript. All authors read and approved the final manuscript.
